# Temporal rich-club phenomenon and its formation mechanisms in international trade: Evidence from new energy minerals

**DOI:** 10.1016/j.isci.2026.114930

**Published:** 2026-02-06

**Authors:** Qianyong Tang, Huajiao Li, Feng An, Yuqi Zhang, Yajie Qi, Xinxin Zheng

**Affiliations:** 1 School of Economics and Management, China University of Geosciences, Beijing 100083, China; 2 MOE Social Science Laboratory of Mineral Resources Security Governance, China University of Geosciences, Beijing 100083, China; 3 The College of Economics and Management, Beijing University of Chemical Technology, Beijing 100029, China; 4 Department of Management, Beijing Electronic Science & Technology Institute, Beijing 100070, China

**Keywords:** Earth sciences, Energy resources, Network modeling, Energy materials

## Abstract

The temporal rich-club (TRC) phenomenon, in which a tight and persistent collection of key nodes is formed, is widely observed in real-world settings. However, the underlying mechanisms that drive the formation of TRC structures remain insufficiently understood. This study investigates the TRC phenomenon in international trade through an analysis of 30 time-evolving trade networks of new energy minerals (cobalt, lithium, nickel, and copper) from 1994 to 2023. We select and weight the features in network evolution using the differentiable information imbalance (DII) method and develop an analytical framework to analyze TRC formation and elucidate its evolutionary mechanism. The empirical results demonstrate statistically significant TRC characteristics in these trade networks. Mathematical modeling reveals that TRC emergence is driven by three coexisting mechanisms, including path dependence, degree of homophily, and intrinsic national attributes such as economic development and resource endowment. These findings provide additional insights into the stability and evolution of global energy mineral trade networks.

## Highlights

• Analysis of new energy mineral trade network evolution from 1994 to 2023
• Incorporating 11 node-level trade attributes
• A scalable DII feature-selection framework for uncovering TRC mechanisms
• Identifying joint drivers of TRC formation

## Graphical abstract

**Figure:**
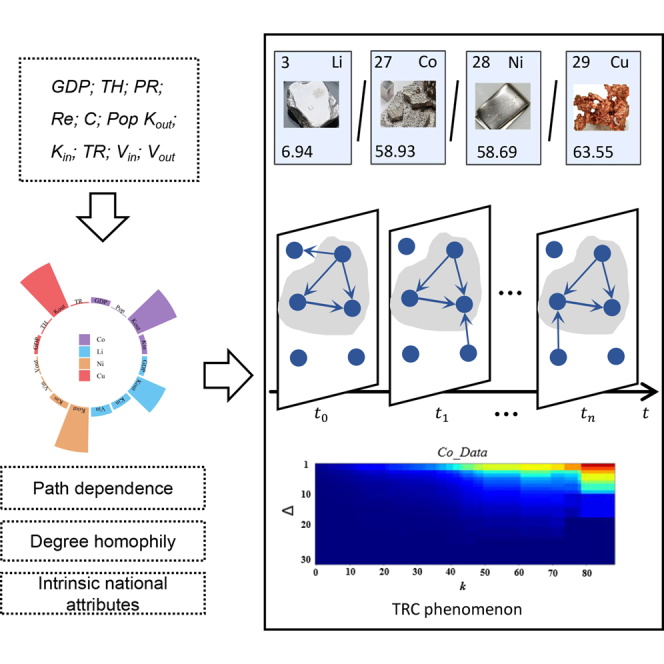

## Introduction

Mineral resources for new energy, such as cobalt, lithium, nickel, and copper, have become essential raw materials for the global energy transition and sustainable development.^1^^,^^2^^,^^3^ As the global demand for low-carbon energy continues to rise, driven by the widespread use of electric vehicles, renewable energy technologies, and energy storage systems, the demand for new energy minerals has soared.^4^^,^^5^ These minerals are not only essential to the clean energy industry but also play pivotal roles in shaping global economic trends, political dynamics, and energy security.^6^ However, the global trade of new energy minerals has exhibited a TRC pattern, which has arisen from the highly uneven global distribution of these resources. A few countries control most reserves, whereas many others rely heavily on imports to satisfy domestic demand. As a result, the trade network for these minerals has become both complex and deeply globalized.^7^^,^^8^ The TRC phenomenon may exacerbate inequalities in access to new energy mineral resources; this not only reduces the efficiency of global resource allocation but also introduces a range of risks for import-dependent countries, including challenges to energy security, supply chain resilience, and geopolitical stability.^9^^,^^10^ In this context, it is crucial to investigate the TRC phenomenon within the global trade network of new energy minerals, with particular attention to the dynamic mechanisms driving its formation and evolution.

The TRC phenomenon refers to a tightly connected and stable subnetwork formed by high-degree nodes in a dynamically evolving network. This pattern highlights not only the structural stability of the network but also the persistent and interdependent nature of resource flows and trade relationships. Early studies on the rich-club effect focused primarily on static network structure.^11^^,^^12^^,^^13^ More recently, research attention has shifted toward temporal networks, with explorations of the manifestation and mechanisms of TRC in dynamic contexts.^14^^,^^15^^,^^16^ The TRC phenomenon has been explored and confirmed in various domains, including social networks,^17^^,^^18^ financial networks,^19^ and brain networks,^20^^,^^21^ and has become a key topic in the study of complex network evolution. However, research on the TRC phenomenon within international trade networks remains limited, particularly in the context of new energy minerals. With accelerating globalization and the rapid rise of the new energy sector, increasing attention is now being given to the presence and implications of TRC in this field.

Studying the evolutionary mechanisms behind the TRC phenomenon is crucial for understanding both the structural stability and functional dynamics of complex networks. Especially in dynamic settings, uncovering how TRC forms can elucidate why certain core nodes maintain long-term dominance. From the simplest preferential attachment models to more complex hybrid evolutionary mechanisms, these theories offer different perspectives for understanding the dynamic evolution of node connectivity within networks.^22^^,^^23^^,^^24^^,^^25^ Current research on the evolution of international trade networks mainly focuses on the temporal changes in trade patterns,^26^^,^^27^ network indicators^28^^,^^29^ (such as degree and betweenness centrality), and network resilience.^30^^,^^31^^,^^32^ These studies primarily describe the outcomes of network evolution rather than how the network evolves, remaining largely at a descriptive results level. Systematic investigations into the underlying mechanisms and the roles of node-level attributes that drive network evolution are still insufficient. Meanwhile, the formation mechanisms of the TRC phenomenon vary across different systems and require in-depth analysis based on the specific conditions of each domain. For example, the evolutionary patterns of social networks and power grids differ significantly, with the former undergoing frequent changes and the latter remaining relatively stable^33^^,^^34^^,^^35^; this indirectly reflects the impact of evolutionary mechanisms on structural differences in networks. In contrast, the evolution of international trade networks is more complex and influenced by a combination of economic, social, and political factors, including resource endowments, geographical location, level of economic development, and international cooperation.^36^^,^^37^^,^^38^^,^^39^ Among these factors, the inherent attributes of nodes play an important role in shaping connection patterns.^40^ These diverse influencing factors indicate that the evolution of international trade networks cannot be explained by a single mechanism alone but rather requires a comprehensive consideration of the combined effects of multiple aspects.^16^ Therefore, although existing network evolution models provide important theoretical support for understanding the formation mechanisms of the TRC phenomenon, a systematic theoretical framework capable of integrating these complex influencing factors to comprehensively explain the evolutionary mechanisms of TRC in international trade networks is still lacking.

A key challenge in constructing a unified theoretical framework to explain the TRC phenomenon lies in performing effective feature selection and weighting among multidimensional influencing factors. To address this, the present study introduces the differentiable information imbalance (DII) method. Originally designed to evaluate the impact of COVID-19 policies,^41^ the method was later adapted by Wild et al. for feature selection and weighting in molecular systems.^42^ Owing to its effectiveness in identifying key features within complex systems, the DII method has been widely applied in various fields, such as the structural characteristics of glass dynamics^43^ and the spread of infectious diseases,^44^ thus providing a solid theoretical foundation for this study.

To uncover the evolutionary mechanisms of the TRC phenomenon in the trade network of new energy minerals, this study applies the DII method to analyze international trade data for cobalt, lithium, nickel, and copper from 1994 to 2023. The analysis incorporates 11 key factors known to influence trade, including GDP, political stability (PR), and resource endowments (Re) (Table 1), to enable effective selection and weighting of relevant features. On this basis, to further characterize the formation process of the TRC phenomenon, this work constructs a temporal feature-weighted evolution (T-FWE) model that integrates path dependence, degree homophily, and intrinsic national attribute mechanisms to analyze their joint effects on the trade network of new energy minerals. This study verifies the explanatory power of the model for the TRC phenomenon and reveals the dynamic process by which connections among high-degree nodes gradually strengthen and stabilize over time. This research not only provides an alternative methodological perspective for analyzing the formation mechanism of the TRC phenomenon but also offers important insights for understanding and ensuring the structural stability and security of new energy mineral resources within global trade networks.

**Table 1:** Features and corresponding data sources

Feature	Description	Reference	Data source
*GDP*(*i*)	Gross domestic product of node *i*.	Jing et al., Huang et al.^45^^,^^46^	World Bank
*TH*(*i*)	Percentage of medium and high-tech exports of node *i* in the manufacturing industry.	Wang et al.^47^	World Bank
*PR*(*i*)	Political risk score of node *i*.	Zhang et al., Zhu et al.^48^^,^^49^	PRS Group
*Re*(*i*)	Resource reserves of node *i*.	Zheng et al.^50^	USGS
*C*(*i*)	Apparent consumption volume of node *i*.	Huang et al., Wang et al.^46^^,^^47^	USGS
*Pop*(*i*)	Population size of node *i*.	Jing et al.^45^	World Bank
*K*_*out*_(*i*)	Number of export partners of node *i*.	Helpman et al.^51^	UN Comtrade
*K*_*in*_(*i*)	Number of import partners of node *i*.	Helpman et al.^51^	UN Comtrade
*TR*(*i*)	Total trade volume of node *i*.	Zhong and Su^52^	UN Comtrade
*V*_*in*_(*i*)	Total import volume of node *i*.	–	UN Comtrade
*V*_*out*_(*i*)	Total export volume of node *i*.	–	UN Comtrade

## Results

### International trade temporal rich-club analysis framework

**Figure 1:**
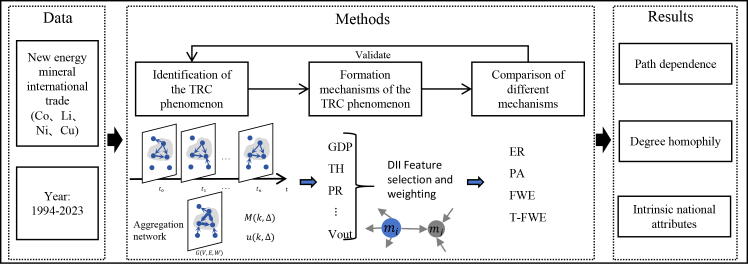
International trade TRC analysis framework

As shown in Figure 1, the international trade TRC analysis framework begins by constructing a temporal network based on global trade flows of new energy minerals from 1994 to 2023. Taking cobalt as an example, each annual network *G*(*t*) = (*V*(*t*), *E*(*t*), *W*(*t*)) represents the trade relationships in year t. The node set *V*(*t*) includes all economies engaged in cobalt trade in year t, and the edge set *E*(*t*) = {*e*_*ij*_(*t*)} denotes directed trade links from economy i to j in year t, and *W*(*t*) = {*w*_*ij*_(*t*)} is the trade volume from node i to j in year t. See the Methods section for detailed information.

Then, using the concept of the TRC, the analysis determines core nodes in the network that are densely interconnected, thereby revealing rich-club characteristics in trade patterns across different minerals. To achieve this, we first calculate the TRC coefficient *M*(*k*,Δ) to determine the presence of the TRC phenomenon. Here, the degree *k* of a node in *G* represents the number of distinct nodes it has interacted with at least once, and its strength *s* denotes the total number of temporal edges it has participated in. Δ denotes the time interval. To verify that the observed TRC phenomenon is not due to randomness, a null model is constructed for comparison. The ratio between the empirical and null results is defined as *u*(*k*,Δ). When *u*(*k*,Δ) ≥ 1, the temporal network is considered to exhibit the TRC phenomenon; otherwise, the phenomenon is absent.^14^^,^^16^ Detailed information can be found in the TRC Phenomenon subsection of the Methods.

On this basis, the study then conducts an in-depth analysis of the evolutionary mechanisms by selecting 11 features with node attributes, including gross domestic product^45^^,^^46^ (GDP), percentage of medium and high-tech exports^47^ (TH), political risk score^48^^,^^49^ (PR), resource reserves^50^ (Re), apparent consumption volume^46^^,^^47^ (C), population size^45^ (Pop), number of export partners^51^ (K_out_), number of import partners^51^ ((K_in_), total trade volume^52^ (TR), total import volume (V_in_), total export volume (V_in_) (Details are provided in Table 1), and considers the influence of these features separately for importing and exporting countries. Then, we use the DII method for feature selection and feature weighting. In addition, the framework compares results from four types of evolutionary models: the Erdős-Rényi model (ER), the preferential attachment model (PA), the feature-weighted evolution (FEW), and the proposed T-FWE model. Their results are validated against empirical trade data. Finally, through the analysis of the results, the framework reveals the evolutionary mechanisms underlying the global trade network of new energy minerals.

### Temporal rich-club phenomenon in new energy minerals

**Figure 2:**
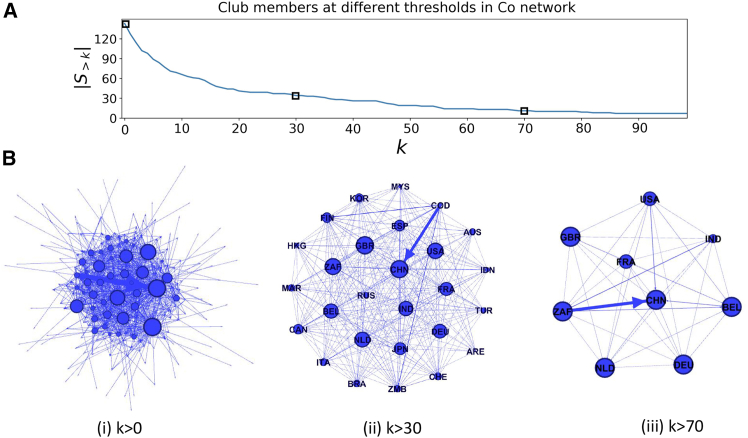
Trend in the number of club members in the aggregated Co network as the threshold increases, and a schematic diagram of the network Selected abbreviations: United States (USA), China (CHN), the Netherlands (NLD), United Kingdom (GBR), South Africa (ZAF), France (FRA), Germany (DEU), India (IND), and Belgium (BEL).

To evaluate the presence of the TRC phenomenon in the international trade of new energy minerals, this study uses the node degree as the indicator of richness. Using cobalt as an example, Figure 2A illustrates how the number of club members changes as the node degree increases in the aggregated network. In networks with *k* > 100, there are only two nodes: the United States and China. When the threshold is lowered to *k* > 70, the club expands to include nine countries: the United States, China, the Netherlands, the United Kingdom, South Africa, France, Germany, India, and Belgium. These countries maintain an average connectivity rate of approximately 30% over short time spans of 2–3 years. In networks with *k* > 30, there are 27 members, representing the core structure of the global cobalt trade network.

**Figure 3:**
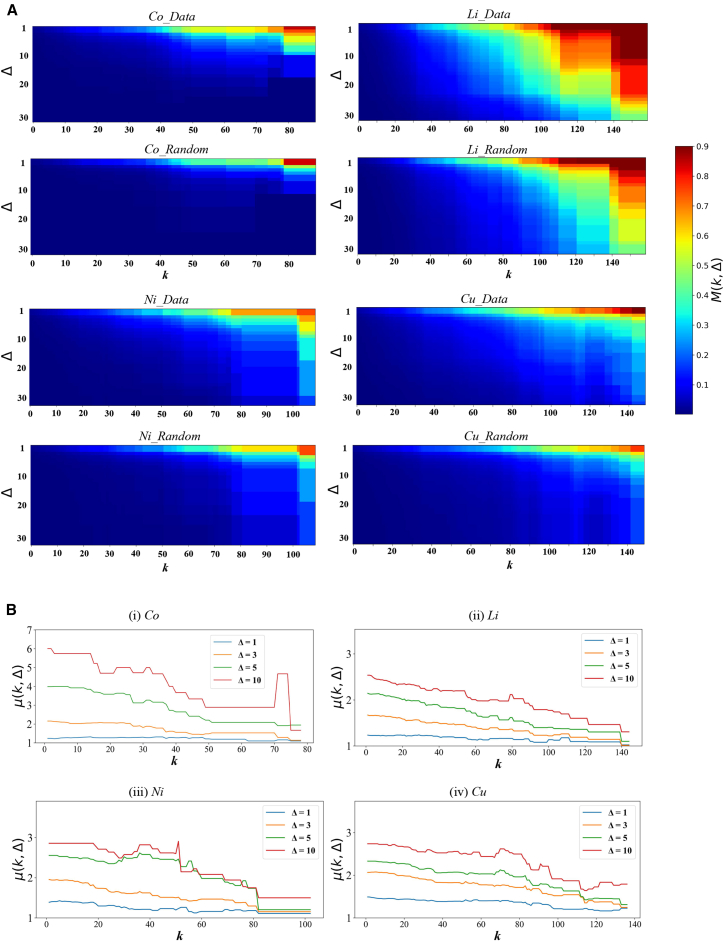
Temporal networks of new energy minerals (A) TRC coefficients of the real trade network and the random network generated by the null model. (B) Ratio of the two for selected time intervals. Note: Cobalt (Co), lithium (Li), nickel (Ni), and copper (Cu).

Figure 3A presents the TRC coefficients *M*(*k*,Δ) of the Co, Li, Ni, and Cu trade networks, along with the corresponding coefficients derived from a null model. For the Co trade network, *M*(*k*,Δ) begins to exhibit a noticeable increase at *k* = 30, with this shift occurring slightly earlier than in the randomized network; this suggests that as the node degree increases, the interconnectivity among high-degree nodes in the Co network becomes increasingly strong and stable. *M*(50,1) approaches 0.5, indicating that the corresponding nodes possess approximately half of all possible interconnections. As the node wealth increases, the connectivity among these nodes becomes progressively denser. An examination of the ratio *u*(*k*,Δ) (Figure 3B) reveals that the Co network consistently has the highest values among the new energy minerals. Notably, when Δ = 10, the ratio for cobalt reaches nearly 7, suggesting that its short-term synchronization and stability are significantly stronger than those of the randomized network. Although the ratio gradually decreases with increasing *k*, cobalt maintains relatively high *u*(*k*,Δ) values, reflecting a pronounced TRC effect across degree thresholds.

In contrast, the *u*(*k*,Δ) values for lithium, nickel, and copper are significantly lower than those for cobalt, particularly under longer temporal windows. Notably, the lithium trade network has the highest rich-club coefficient, with *M*(140,10) approaching 0.9. However, the corresponding coefficient in the randomized network is also relatively high, indicating that the most central nodes (such as China, the United States, and Germany) maintain long-term stable trade relationships. Specifically, 35 bilateral trade links among these countries persist annually, and randomization does not substantially affect the connectivity pattern. Notably, across different energy mineral networks and for various values of Δ, the condition u(k,Δ) > 1 consistently holds, indicating that all of these trade networks exhibit clear evidence of the TRC phenomenon.

### Evolutionary mechanism of the temporal rich-club in new energy minerals

**Figure 4:**
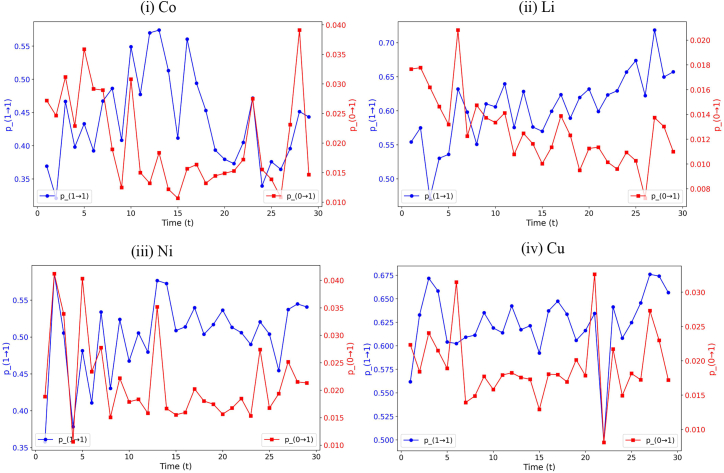
Network changes in Co, Li, Ni, and Cu from time *t*-1 to *t* *p*_(1→1) represents the probability that a trade link survives from *t*-1 to *t*; *p*_(0→1) represents the probability of a new trade link birth from *t*-1 to *t*. *t* = 1 corresponds to the year 1995, *t* = 2 to 1996, and so on.

Figure 4 illustrates the network changes in the temporal trade networks of cobalt, lithium, nickel, and copper from time *t*-1 to *t* (where t = 1 refers to the transition from the 1994 network to the 1995 network). In each panel, the blue line represents the probability *p*_(1→1), indicating the probability that a trade link survives from *t*-1 to *t*. The red line represents *p*_(0→1), the probability of a new trade link birth in the same period. The plots reveal varying degrees of volatility in both probabilities across different products. For instance, the probability of new link formation *p*_(0→1) exhibits notable fluctuations at multiple time points across all four commodities. Notably, the average persistence probability *p*_(1→1) for lithium and copper remains above 0.6, whereas that for cobalt and nickel is approximately 0.5. In contrast, cobalt and nickel also show greater fluctuations in the probability of link disappearance. This disparity may be attributed to differences in network size: the trade networks for copper and lithium are substantially larger than those for cobalt and nickel, which may contribute to higher network stability and more persistent linkages in the former.

Network evolution is influenced by multiple factors. To further uncover the mechanisms behind TRC formation, the DII method is employed to perform feature selection and weighting on 11 node-level attributes. In directed trade networks, link formation is not only driven by exporter characteristics but also closely related to importer features.

**Figure 5:**
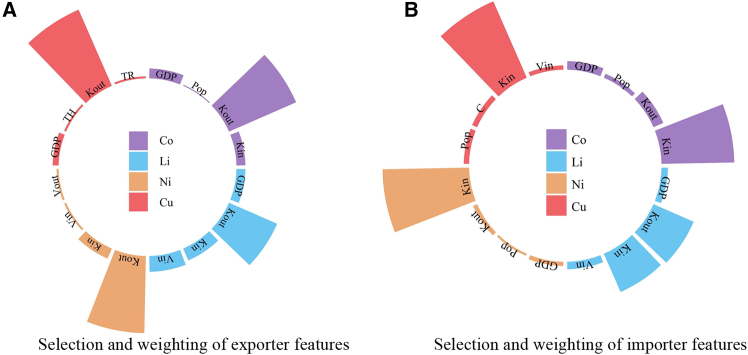
Features and weights of import and export countries influencing the stability of high-density trade connections

Figure 5 presents the results of feature selection and weighting, where the bar height indicates the relative importance (weight) of each feature. The top four features are retained, while the weights of all other variables fall below 0.1, suggesting limited influence. For exporter features (Figure 5A), the number of export trade partners (out-degree) plays a pivotal role in contributing to the density and stability of the trade network, receiving the highest weight. Interestingly, the number of import partners also appears to be a key exporter feature, possibly due to the unequal distribution of energy mineral resources: a small number of countries possess abundant reserves, while many others function as intermediary hubs. In addition, GDP is a common influential factor across all four commodities, indicating that economic size significantly contributes to the formation of dense and stable export structures.

For importer features (Figure 5B), the number of import trade partners (in-degree) plays a critical role in determining the density and stability of the trade network for cobalt, nickel, and copper, which receive the highest feature weight. In contrast, for lithium-importing countries, the most influential attribute is the number of export partners (out-degree), followed by the in-degree. In addition, the feature set for lithium-importing countries also includes GDP and total import volume. The apparent consumption of copper is identified as an important characteristic of copper-exporting countries. Moreover, node attributes such as population also influence the sustained stability of exports to some extent.

### Model validation

**Figure 6:**
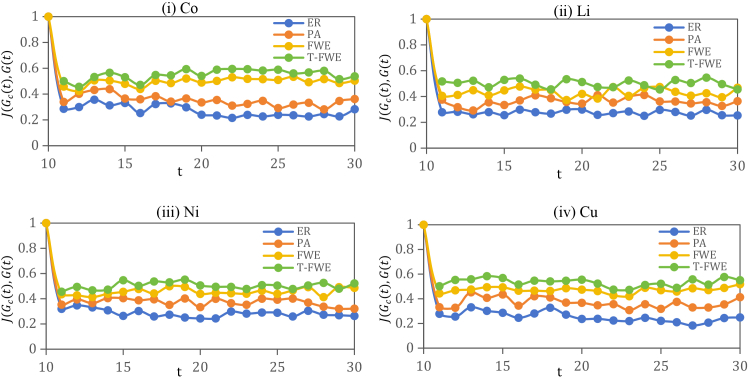
Jaccard similarity index between network structures predicted by different models and the real network

To validate the effectiveness of the model, we compare four network evolution models: ER, PA, FWE, and T-FWE. To reduce stochastic effects, all the results are averaged over 10 independent simulation runs. The prediction accuracy is evaluated via the Jaccard similarity coefficient between the predicted and actual trade networks, as shown in Figure 6. In the T-FWE model, predictions for year *t*+1 are made based on data from the previous ten years. For example, data from 1994 to 2003 are used to predict the network in 2004. Accordingly, the similarity *J*(*G*_*c*_(*t*),*G*(*t*)) = 1 in 2003. The results show that the ER model yields approximately 30% similarity with the actual network, whereas the T-FWE model consistently maintains a similarity above 50%. Across all four commodities, the prediction accuracy ranks consistently as ER < PA < FWE < T-FWE, with T-FWE demonstrating a clear advantage in similarity performance.

**Figure 7:**
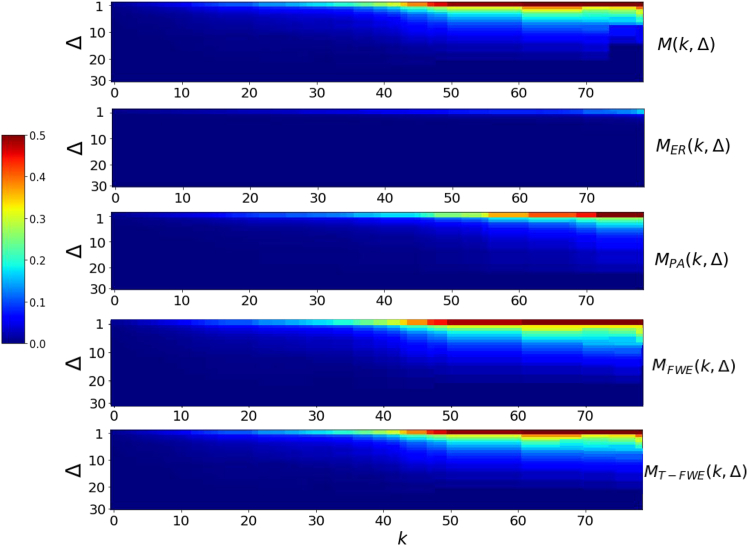
TRC coefficients generated by different models for the cobalt trade network

To further validate the existence of the TRC phenomenon, we apply different evolution models to the cobalt trade network and compute their respective TRC coefficients. Figure 7 compares the TRC coefficients of networks generated by different models with those of the empirical network. The core structure of the random network is entirely stochastic and lacks stability. A significant gap also exists between *M*_*PA*_(*k*,Δ) and the empirical *M*(*k*,Δ), indicating that the preferential attachment mechanism alone is insufficient to account for the emergence of TRC patterns. In contrast, the TRC coefficients computed under the T-FWE model, *M*_*T*-*FWE*_(*k*,Δ), incorporate temporal path dependence, allowing the model to capture the influence of exogenous temporal shocks, such as economic crises, geopolitical events, and demographic disruptions, that may lead to the collapse of rich-club structures. Furthermore, the model accounts for node-level heterogeneity, particularly the differing roles of countries in export and import dynamics. For example, certain transit countries exhibit structural advantages in trade due to their inherent positional characteristics.

### Sensitivity analysis

**Figure 8:**
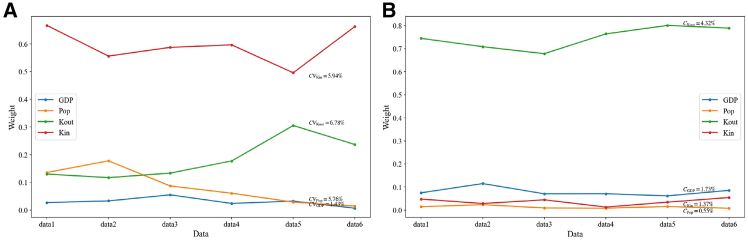
Variations in feature weights and their coefficients of variation for cobalt trade under different temporal windows (the left panel shows importer feature weights, and the right panel shows exporter feature weights) Data from 1994 to 2008 were used as data1, and subsequent temporal windows were constructed using a sliding-window approach, with the period 1997–2011 serving as the second window. By shifting the window by three years each time, six datasets (data1-data6) were generated in total.

To validate the effectiveness of the DII-based feature weighting, the temporal trade network of cobalt was divided into datasets corresponding to different time windows. Data from 1994 to 2008 were used as data1, and subsequent temporal windows were constructed using a sliding-window approach, with the period 1997–2011 serving as the second window. By shifting the window by three years each time, a total of six datasets (data1-data6) were generated. This design ensures that the temporal windows vary over time, while the first and last datasets represent completely distinct time periods. The feature weights for each dataset were calculated, and the coefficients of variation were derived across all datasets to measure the variability of these feature weights. The coefficient of variation is defined as *CV*_*i*_ = *σ*_*i*_/*φ*_*i*_, where *CV*_*i*_ denotes the coefficient of variation for feature *i*, *σ*_*i*_ represents the standard deviation of the feature’s weight across different time windows, and *φ*_*i*_ is the mean weight of that feature. A smaller value indicates that the feature weight varies little across time windows, implying high robustness.^53^^,^^54^ As shown in Figure 8, the variations in the selected features (GDP, Pop, Kout, and Kin) were calculated. The results show that all features have *CV* < 0.1, indicating that the DII-based feature weights are highly stable across different temporal windows and that the feature importance remains consistent.

**Figure 9:**
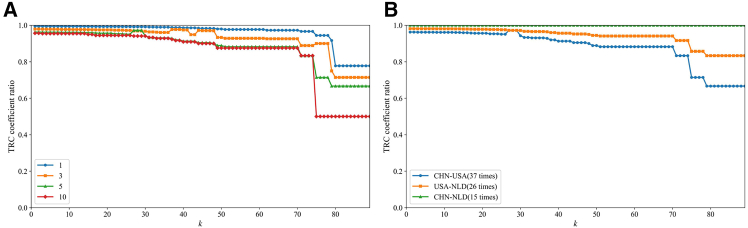
The cobalt network after the removal of the key national linkage (A) The key national linkage (China-United States) is used to simulate the impact of geopolitical risks on the TRC coefficient (temporal intervals Δ = 1, 3, 5, and 10). The vertical axis represents the ratio of the TRC coefficient of the perturbed network to that of the original network. (B) The effect of removing core connections (CHN-USA, USA-NLD, and CHN-NLD) on the TRC ratio under a temporal interval of Δ = 5. These three cores directed trade links appear 37, 26, and 15 times, respectively, over the sample period.

As shown in Figure 9A, to examine the sensitivity of the TRC coefficient to key linkages, we simulate a geopolitical shock by removing the bilateral trade connection between China and the United States. The TRC ratios of the perturbed network to the original network are then computed under different temporal intervals Δ (1, 3, 5, and 10). When the threshold *k* is small, the ratios remain close to 1, reflecting the presence of a relatively large set of rich nodes; as *k* increases and the core group becomes smaller, the effect of link removal becomes increasingly pronounced. Mathematically, since removing a single edge leads to $|E_{>k}^{\prime }\left|=\right|E_{>k}-1|$, the resulting change in the TRC coefficient can be written as Δ*TRC* = 2/(|*N*_>*k*_|(|*N*_>*k*_|-1). This expression clearly shows that the variation in Δ*TRC* becomes substantial only when the number of rich nodes is relatively small. The size-dependent resilience implied by this result reflects a lock-in effect arising from the accumulated history of trade interactions: as repeated exchanges reinforce the ties among a sufficiently large set of core countries, the rich-club structure becomes increasingly stabilized, and the removal of any single link produces only minimal perturbation.

Building on this insight, Figure 9B further examines how path dependence shapes the response of the rich-club to geopolitical disruptions. We remove the three most frequently occurring bilateral links among the highest-degree core countries in the cobalt network (CHN-USA, USA-NLD, and CHN-NLD), which appear 37, 26, and 15 times, respectively, over the sample period. Using Δ = 5, we compute the TRC ratio after each link removal. The results show that deleting more frequently observed links leads to a noticeably larger decline in the TRC coefficient. Because these ties have been reinforced over long periods, they possess greater structural embeddedness within the rich-club, and their removal disrupts not only current connectivity but also the historical foundations on which the core structure is built. This indicates that the network’s vulnerability or resilience to geopolitical shocks is determined by the same path-dependent dynamics that stabilize the rich-club during normal periods.

## Discussion

This study builds an effective network evolution model based on 30 years of trade data (1994–2023) to simulate the TRC phenomenon in international trade networks of new energy minerals, including cobalt, lithium, nickel, and copper. By capturing the dynamic structure of these networks, the model improves the prediction of trade pattern evolution and provides a solid foundation for promoting the stability and sustainable development of the global energy supply system.

The trade networks of cobalt, lithium, nickel, and copper all exhibit clear TRC phenomena. As the node degree increases, the connections among high-degree nodes grow progressively stronger and more stable. Among the four commodities, the TRC effect is most pronounced in the cobalt trade network, especially within shorter time windows, where its synchronization and stability significantly exceed those of the other mineral networks. In contrast, the lithium trade network has the highest local TRC coefficients, indicating that its core nodes are not only densely connected but also exhibit a high degree of temporal stability. These core nodes are typically major producers, consumers, or transit hubs of lithium, such as China, the United States, and Germany, whose trade relationships have remained consistently aligned over extended periods.

This study identifies three primary mechanisms underlying the formation of TRC structures. The first is path dependence, which suggests that the longer a trade relationship persists, the more stable the network connection becomes. This is consistent with the findings by Li et al. regarding the temporal dependence mechanism in international fertilizer trade networks.^16^ This mechanism can be explained through factors such as communication costs, comparative advantage, and economies of scale.^51^^,^^55^^,^^56^ Specifically, as time progresses, countries engaged in trade tend to establish increasingly stable relationships, which are reinforced by previous interactions and accumulated trade history. Long-term trade cooperation can reduce transaction costs, enhance the efficiency of information flow, and further reinforce comparative advantages between countries—ultimately fostering mutually beneficial relationships. The geopolitical shock analysis presented earlier further illustrates this mechanism, showing that links with deeper historical reinforcement exert a disproportionately strong influence on the stability of rich-club structures.

The second mechanism is degree homophily, which suggests that the most influential feature weights for both exporting and importing countries are generally correlated with node degree. Specifically, nodes with high export degrees tend to direct trade flows toward nodes with high import degrees. This pattern can be explained by demand‒supply dynamics and reputational attractiveness, reflecting the homophilic nature of node interactions within the network. Countries with high export degrees are often those with resource or geographic advantages, and they tend to trade with countries that exhibit similarly strong demand or have large markets, thereby establishing more stable trade relationships. This mechanism is closely aligned with preferential attachment theory in complex networks and the gravity model of trade in economics.^57^

The mechanisms of path dependence and degree homophily have been empirically verified across various types of networks, including international trade networks, spatial trade and production network and social networks.^16^^,^^58^^,^^59^^,^^60^ The third mechanism is intrinsic national attributes, which refer to the inherent characteristics of nodes that influence the formation and evolution of trade relationships, such as the level of economic development and resource endowment. These attributes shape a country’s competitiveness and attractiveness within the global trade network, thereby affecting its position and the stability of its trade connections. For example, countries with higher levels of economic development typically possess greater production capacity and consumption demand, enabling them to attract a larger number of trade partners.^51^ In contrast, resource-rich countries often serve as key nodes in global supply chains, especially in the trade of strategic commodities such as critical energy minerals. These intrinsic factors not only influence trade flow directions between countries but also determine the strength and persistence of linkages within the network.

Based on the above evolutionary mechanisms, we propose the following policy recommendations.

1. The temporal rich-club coefficient allows the identification of core countries in international trade networks. Both exporting and importing countries should closely monitor the trade policy changes of these core countries and adjust their own trade strategies in a timely manner. While intrinsic national attributes such as resource endowment and the level of economic development are relatively immutable in the short term, the adaptive mechanisms of path dependence and degree of homophily can be influenced through policy interventions. Therefore, targeted strategies focusing on these mechanisms can help enhance trade security and improve the resilience of global supply chains.
2. Under the path dependence mechanism, long-term trade relationships contribute to network stability but may also lead to excessive concentration and systemic vulnerability. Resource-rich exporters should maintain stable partnerships to consolidate credibility while avoiding overreliance on a few major partners by diversifying markets and strengthening regional cooperation. Import-dependent countries, in turn, should reduce single-source dependency by developing diversified import channels, establishing strategic reserves, and creating alternative supply arrangements to mitigate potential disruptions. The geopolitical shock analysis indicates that historically reinforced links play an outsized role in sustaining core structures, suggesting that policymakers should closely monitor such high-dependence relationships as part of risk assessment and early-warning strategies.
3. Under the degree homophily mechanism, attention should be given to improving the structural inclusiveness of trade networks. Excessive clustering among high-degree nodes reinforces inequality and heightens systemic risks. Exporters can promote balanced network development by engaging more with medium- and low-degree countries, thereby diffusing centrality and stabilizing global trade flows. Importers can strengthen trade relations with emerging suppliers and regional partners to improve inclusiveness, reduce structural vulnerability, and foster a more sustainable and equitable global trading environment.

### Limitations of the study

This study proposes a framework for analyzing the mechanisms underlying TRC structures by applying the DII method to select and weight features that influence TRC formation during the evolution of trade networks. While this approach provides additional insights into the structural dynamics of international trade, it does not fully capture the complexity of real-world trade systems. In particular, external factors such as trade policies and tariff adjustments may significantly influence the formation and dissolution of trade relationships. These aspects were not explicitly incorporated into the present model. Future research could integrate such factors to enhance the model’s realism and predictive power.

## Resource availability

### Lead contact

Requests for further information and resources should be directed to and will be fulfilled by the lead contact, Huajiao Li (babyproud@126.com).

### Materials availability

This study did not generate new unique reagents.

### Data and code availability

• This article analyzes existing, publicly available data. These accession numbers for the datasets are listed in the key resources table.
• All original code used in this study, including the primary software and algorithms, is listed in the key resources table.
• Any additional information required to reanalyze the data reported in this article is available from the lead contact upon request.

## Acknowledgments

This research is supported by grants from the Deep Earth Probe and Mineral Resources Exploration - the 10.13039/501100018537National Science and Technology Major Project (Grant No. 2025ZD1007004), the 10.13039/501100001809National Natural Science Foundation of China (Grant Nos. 72173119, 71991481, and 71991480), the Fundamental Research Funds for the Central Universities (Grant Nos. 2652019087 and 2652019241), and the Key Projects of Beijing Social Science Foundation (Grant No. 22JCB051). Meanwhile, the authors would like to thank AJE (American Journal Experts) for their professional suggestions about the language usage, spelling, and grammar of this article.

## Author contributions

Q.T., writing - original draft, visualization, methodology, formal analysis, and data curation. H.L., writing – review and editing, supervision, resources, funding acquisition, and conceptualization. F.A., results review and editing. Y.Z., results review and editing. Y.Q., reviewing and editing. X.Z., reviewing and editing.

## Declaration of interests

The authors declare no competing interests.

## STAR★Methods

### Key resources table

**Table:** 

REAGENT or RESOURCE	SOURCE	IDENTIFIER
**Deposited data**		

New energy mineral international trade data	United Nations Comtrade Database	https://comtradeplus.un.org/
A country’s GDP and the percentage of medium- and high-technology exports	World Bank	https://data.worldbank.org.cn/
A country’s political risk	PRS Group	https://www.prsgroup.com/
A country’s resource reserves and apparent consumption	USGS	https://www.usgs.gov/

**Software and algorithms**		

Python	Python Software Foundation	https://www.python.org/
Jupyter Notebook	Project Jupyter	https://jupyter.org/
DADApy	Python package	https://dadapy.readthedocs.io/en/latest/
NetworkX	Python package	https://networkx.org/en/
The algorithms involved in the results of this paper	N/A	https://github.com/Tmelo770/IR-TRC

### Experimental model and study participant details

There are no experimental model or study participants to include in this study.

### Method details

#### Data processing

This study utilizes trade data from the United Nations Comtrade Database and focuses on four representative new energy minerals: cobalt ore, lithium ore, nickel ore, and copper ore. For each mineral, a temporal trade network is constructed based on 30 years of trade flow data spanning from 1994 to 2023. The country codes follow the ISO 3-digit country abbreviation system. In year *t*, the trade flow between country *i* and country *j* is denoted as *w*_*ij*_(*t*), measured in kilograms. For each trade flow, two records are typically recorded, i.e., one from the importing country *j* and another from the exporting country *i*. However, due to potential data incompleteness, these two records may not always align perfectly. To address this, *w*_*ij*_(*t*) is defined as the larger of the two values, representing the trade flow from country *i* to country *j* at a given point in time in the international trade network. In addition, several country-level characteristics are incorporated in this study. To ensure comparability across features with different units and scales, all variables are standardized via *Z* score normalization.

#### TRC phenomenon

Temporal Network Construction. This study builds a series of temporal international trade networks for new energy minerals. Taking cobalt as an example, each network consists of nodes (economies), links (trade flows), and weights (trade volume). We define each year from 1994 to 2023 as a subnetwork, forming 30 annual temporal networks, which are denoted as *G*(*t*)=(*V*(*t*),*E*(*t*),*W*(*t*)). Here, the node set *V*(*t*) = {*v*(*t*)} represents all economies involved in cobalt trade in year t, and the edge set *E*(*t*) = {*e*_*ij*_(*t*)} represents the trade relationships from economy *i* to economy *j* in year t. If a trade relationship exists at time t, then *e*_*ij*_(*t*) = 1; otherwise, it is 0. The edge set *W*(*t*) = {*w*_*ij*_(*t*)} represents the trade volume from node i to j in year t. Thus, we construct a directed weighted temporal network.

TRC coefficient *M*(*k*,Δ). According to the definition by Pedreschi et al., the TRC phenomenon refers to the emergence of a tightly connected and stable subnetwork formed by high-degree nodes in a dynamically evolving network.^14^ The TRC coefficient *M*(*k*,Δ) is defined as:

(Equation 1) $$\begin{array}{c} M\left(k,\Delta \right)=\underset{t}{\max\;}\epsilon_{>k}\left(t,\Delta \right) \end{array}$$

where *k* denotes the degree of a node and where Δ denotes the time interval. As Δ increases, *M*(*k*,Δ) reflects the tendency of the richest nodes to form synchronized and stable interconnections over at least the duration of Δ. The term *ϵ*_>*k*_(*t*,Δ) denotes the local TRC coefficient in the temporal network, which is defined as follows^14^:

(Equation 2) $$\begin{array}{c} \epsilon_{>k}\left(t,\Delta \right)=\frac{\left|E_{>k}\left(t,\Delta \right)\right|}{\left|V_{>k}\right|\left(\left|V_{>k}\right|-1\right)} \end{array}$$

where the notation ∣⋅∣ denotes the number of elements in a set. |*V*_>*k*_| denotes the number of nodes whose degrees exceed a given richness threshold *k*. The numerator |*E*_>*k*_(*t*,Δ)| represents the number of links among these nodes that remain stable throughout the time window [*t*,*t*+Δ], normalized by its maximal possible number of links |*V*_>*k*_|(|*V*_>*k*_|-1).^14^

To determine whether the interconnections among high-degree nodes exceed random effects, it becomes imperative to construct a null model to calculate *M*_*rnd*_(*k*,Δ) and compare it with the actual value *M*(*k*,Δ). In the null model, a reshuffling procedure with parameter *μ* is used to randomly reassign the timestamps of all temporal links while preserving node activity and path structures.^61^ The ratio is calculated as follows:

(Equation 3) $$\begin{array}{c} u\left(k,\Delta \right)=\frac{M\left(k,\Delta \right)}{M_{rnd}\left(k,\Delta \right)} \end{array}$$

When *u*(*k*) > 1, the TRC phenomenon is considered to exist. To enhance the robustness of this assessment, a statistical hypothesis test is conducted to evaluate the significance of the observed TRC.^16^^,^^62^ The null hypothesis assumes that *u*(*k*,Δ) ≤ 1. The *p* value is calculated as follows:

(Equation 4) $$\begin{array}{c} p=\frac{\tau \left(u\left(k,\Delta \right)\leq 1\right)}{n} \end{array}$$

where τ(*u*(*k*,Δ) ≤ 1) denotes the number of times that *u*(*k*,Δ) ≤ 1. The number of simulations n is set to 500 in this study. The smaller the *p* value is, the stronger the evidence against the null hypothesis, and the more favorable it is to the alternative hypothesis that supports the existence of the TRC phenomenon. When *p* < 1%, the TRC phenomenon is considered statistically significant. For systems that pass the statistical test, the average value $\hat{u}\left(k,\Delta \right)$ is used as the final ratio. A higher $\hat{u}\left(k,\Delta \right)$ indicates a stronger TRC phenomenon.

#### T-FWE model

Network evolution model. The essence of network evolution lies in the transformation process from *G*(1), *G*(2), …, *G*(*t*). In the network evolution model proposed in this study, the set of nodes V remains unchanged from *G*(*t*) to *G*(*t*+1), whereas the set of edges is updated as *E*(*t*+1) = *e*_*ij*_(*t*+1) from *E*(*t*). The evolution process is as follows:

(Equation 5) $$\begin{array}{c} e_{ij}\left(t+1\right)=e_{ij}\left(t\right)H\left(\vartheta ,\varphi_{t}^{s}\gamma_{ijt}\Pi_{t}\left(i,j\right)\right)+\left(1-e_{ij}\left(t\right)\right)H\left(\vartheta ,\varphi_{t}^{b}\Pi_{t}\left(i,j\right)\right) \end{array}$$

where *H*(*ϑ*,∂) denotes the Heaviside step function, activated under the following condition:

(Equation 6) $$\begin{array}{c} H\left(\vartheta ,\partial \right)=\left\{ \begin{array}{c} 1,if\partial \geq \vartheta \\ 0,if\partial <\vartheta \end{array}\right. \end{array}$$

The parameter *ϑ* is a randomly drawn value within the range [0, 1], which is independently re-sampled in each simulation run to introduce stochasticity into the results. Multiple simulation iterations are conducted to ensure the robustness and reliability of the outcomes. In the expressions $\varphi_{t}^{s}\gamma_{ijt}\Pi_{t}\left(i,j\right)$ and $\varphi_{t}^{b}\Pi_{t}\left(i,j\right)$, $\varphi_{t}^{s}$ denotes the survival rate of existing links, whereas $\varphi_{t}^{b}$ denotes the birth rate. *γ*_*ijt*_ is the temporal path dependence probability, and *Π*_*t*_(*i*,*j*) is the connection probability between nodes *i* and *j* at time t. The temporal path dependence probability reflects the notion that the longer a link has persisted, the more likely it is to remain active in the next time period.^63^^,^^64^

To ensure that the network evolution in the model closely reflects the pace of change observed in real-world networks, $\varphi_{t}^{s}$ and $\varphi_{t}^{b}$ are defined as follows:

(Equation 7) $$\begin{array}{c} \varphi_{t}^{s}={\bar{\rho }}_{1\to 1}\left(t\right)\frac{|E\left(t\right)|}{\sum_{E\left(t\right)}\Pi_{t}\left(i,j\right)} \end{array}$$

(Equation 8) $$\begin{array}{c} \varphi_{t}^{b}={\bar{\rho }}_{0\to 1}\left(t\right)\frac{|E\left(t\right)|}{\sum_{E\left(t\right)}\Pi_{t}\left(i,j\right)} \end{array}$$

Since the structural changes of the network at time t+1 are not observable in the data, the model approximates the network evolution speed using the average over the observed period from time 1 to t, expressed as:

(Equation 9) $$\begin{array}{c} {\bar{\rho }}_{1\to 1}\left(t\to t+1\right)=\frac{1}{t}\left(\rho_{1\to 1}\left(1\right)+\cdots +\rho_{1\to 1}\left(t\right)\right) \end{array}$$

(Equation 10) $$\begin{array}{c} {\bar{\rho }}_{0\to 1}\left(t\to t+1\right)=\frac{1}{t}\left(\rho_{0\to 1}\left(1\right)+\cdots +\rho_{0\to 1}\left(t\right)\right) \end{array}$$

The proportions of link survival and birth from *G*(*t*) to *G*(*t*+1) are calculated as follows:

(Equation 11) $$\begin{array}{c} \rho_{1\to 1}\left(t\right)=\frac{|E\left(t\right)\cap E\left(t+1\right)|}{|E\left(t\right)|} \end{array}$$

(Equation 12) $$\begin{array}{c} \rho_{0\to 1}\left(t\right)=\frac{|E\left(t+1\right)-E\left(t\right)\cap E\left(t+1\right)|}{\left|V_{>k}\right|\left(\left|V_{>k}\right|-1\right)-\left|E\left(t\right)\right|} \end{array}$$

Calculation of *Π*_*t*_(*i*,*j*). Nodes with higher out-degrees are more likely to connect to nodes with higher in-degrees, a phenomenon widely known as preferential attachment.^65^^,^^66^ The corresponding mathematical assumption is that the probability of a trade relationship occurring between node i and node j is proportional to the product of the out-degree and in-degree, as follows:

(Equation 13) $$\begin{array}{c} \Pi_{t}^{PA}\left(i,j\right)∼k_{i\left(t\right)}^{PA}k_{j\left(t\right)}^{PA} \end{array}$$

In practice, international trade networks exhibit far more complex patterns than those captured by basic preferential attachment. Factors such as the resource endowments of exporters and the demand-side characteristics of importers play a significant role. Thus, the probability of link formation is not solely determined by the node degree but also shaped by specific node-level attributes (e.g., export and import features). To reflect this, Equation 13 is generalized into a feature-weighted model, as shown below:

(Equation 14) $$\begin{array}{c} \Pi_{t}^{FWE}\left(i,j\right)∼m_{i\left(t\right)}^{T-FWE}m_{j\left(t\right)}^{T-FWE} \end{array}$$

where $m_{i\left(t\right)}^{T-FWE}$ and $m_{j\left(t\right)}^{T-FWE}$ denote the feature-weighted aggregations for the exporter and importer nodes, respectively. Their detailed expressions are as follows:

(Equation 15) $$\begin{array}{c} m_{i\left(t\right)}^{T-FWE}=\sum_{z=1}^{Z}\omega_{i\left(z\right)\left(t\right)}^{T-FWE}f_{i\left(z\right)\left(t\right)}^{T-FWE} \end{array}$$

(Equation 16) $$\begin{array}{c} m_{j\left(t\right)}^{T-FWE}=\sum_{r=1}^{R}\omega_{j\left(r\right)\left(t\right)}^{T-FWE}f_{i\left(r\right)\left(t\right)}^{T-FWE} \end{array}$$

where *z* denotes the number of features associated with the exporter node and where *r* represents the number of features associated with the importer node.

ER model. Each edge is randomly removed or generated with a certain probability,^67^^,^^68^ similar to the Erdős–Rényi (ER) model. The node set *V* remains constant, while the link set is updated according to $E^{ER}\left(t\right)=\{e_{ij}^{ER}\left(t\right)\}$. The evolution process of all links can be described as follows:

(Equation 17) $$\begin{array}{c} {e_{ij}}^{ER}\left(t+1\right)=e_{ij}^{ER}\left(t\right)H\left(\vartheta ,{\bar{\rho }}_{1\to 1}\right)+\left(1-e_{ij}^{ER}\left(t\right)\right)H\left(\vartheta ,{\bar{\rho }}_{0\to 1}\right) \end{array}$$

PA model. Nodes with higher out-degrees are more likely to connect to nodes with higher in-degrees, a phenomenon widely known as preferential attachment.^65^^,^^66^ According to Equation 13, the link evolution process can be written as follows:

(Equation 18) $$\begin{array}{c} {e_{ij}}^{PA}\left(t+1\right)=e_{ij}^{PA}\left(t\right)H\left(\vartheta ,\varphi_{t}^{s}\Pi_{t}^{PA}\left(i,j\right)\right)+\left(1-e_{ij}^{ER}\left(t\right)\right)H\left(\vartheta ,\varphi_{t}^{b}\Pi_{t}^{PA}\left(i,j\right)\right) \end{array}$$

Similarly, the FEW model is derived from Equation 5 by removing the time-dependent coefficient *γ*_*ijt*_. The networks generated by different evolution models are then compared with the actual networks to assess model performance.

#### DII feature selection and weighting

Given that different international trade commodities are affected by distinct factors, this study selects node-level features related to national trade characteristics. The differential information imbalance (DII) method is applied for feature selection and weighted aggregation.^42^ According to the definition of the TRC, high-degree nodes in the network gradually form a denser and more stable subnetwork over time. Therefore, in the process of feature selection and aggregation, the product of two rich-club features (degree ∗ stability) is used as the dependent variable to form the target space *d*^*B*^, while the independent variables are selected from among the national attribute factors in international trade to form the feature space *d*^*A*^. Here, we use the sum of the probabilities of a node’s links appearing in the temporal network as its stability.

To identify the most relevant features and optimize their contributions, a weight parameter *ω*_*D*_ is assigned to each feature. This allows the impact of each feature on the distance metric to be scaled based on its importance. The computation is based on the weighted Euclidean distance:

(Equation 19) $$\begin{array}{c} d_{ij}^{A}\left(w\right)=\left|\left|\omega_{1}f_{1i}-\omega_{1}f_{1j},\ldots ,\omega_{D}f_{Di}-\omega_{D}f_{Dj}\right|\right| \end{array}$$

To optimize the information imbalance measure, we apply the Softmax function to convert the ranking operation into a differentiable form. This transformation renders the distance metric smooth and continuous, enabling effective optimization of feature weights via standard gradient descent methods.

(Equation 20) $$\begin{array}{c} DII\left(d^{A}\left(\omega \right)\to d^{B}\right)=\frac{2}{N^{2}}\sum_{i,j=1\left(j\neq i\right):}{c_{ij}\left(\lambda ,d^{A}\left(\omega \right)\right)r}_{ij}^{B} \end{array}$$

where *c*_*ij*_(λ,*d*^*A*^(*ω*)) is the Softmax coefficient computed based on the weighted distance, which represents the similarity between data points *i* and *j* in the weighted feature space *d*^*A*^(*ω*). It is defined as cij(λ,dA(ω))=e−(dijA(w)λ)∑m≠i−(dimA(w)λ), where λ is a smoothing parameter that determines the sensitivity of the similarity function to distance variations. By optimizing the feature weights *w*, the goal is to make the distances in the feature space *d*^*A*^ as close as possible to those in the target space *d*^*B*^, thereby identifying the most explanatory and relevant features.

### Quantification and statistical analysis

All quantitative analyses in this study were conducted using a Python-based computational environment. The primary libraries employed include pandas, NetworkX, and DADApy etc, for data processing, network construction, and feature selection and weighting, respectively. In addition, the null model based on randomized temporal link timestamp generation, the *Z* score normalization of node-level features, and the comparative analysis of different evolutionary models were all implemented in Python. The complete code used in this study is publicly available on GitHub at https://github.com/Tmelo770/IR-TRC.

### Additional resources

There are no additional resources to include in this study.
